# Clinical-Grade Human Embryonic Stem Cell-Derived Mesenchymal Stromal Cells Ameliorate the Progression of Osteoarthritis in a Rat Model

**DOI:** 10.3390/molecules26030604

**Published:** 2021-01-24

**Authors:** Dan Xing, Kai Wang, Jun Wu, Yu Zhao, Wei Liu, Jiao Jiao Li, Tingting Gao, Deng Yan, Liu Wang, Jie Hao, Jianhao Lin

**Affiliations:** 1 Arthritis Clinic & Research Center, Peking University People’s Hospital, Peking University, Beijing 100044, China; lovetroy@126.com (D.X.); wangkai_b4@163.com (K.W.); zhaoyu20806@163.com (Y.Z.); 2 Arthritis Institute, Peking University, Beijing 100044, China; 3 National Stem Cell Resource Center, Institute of Zoology, Chinese Academy of Sciences, Beijing 100190, China; wuxf@ioz.ac.cn (J.W.); gaotting@ioz.ac.cn (T.G.); m17649621579@163.com (D.Y.); wangliu@ioz.ac.cn (L.W.); 4 State Key Laboratory of Stem Cell and Reproductive Biology, Institute of Zoology, Chinese Academy of Sciences, Beijing 100101, China; 5 Institute for Stem Cell and Regeneration, Chinese Academy of Sciences, Beijing 100101, China; 6 Beijing Institute for Stem Cell and Regenerative Medicine, Beijing 100101, China; 7 Department of Biomedical Engineering, School of Medicine, Collaborative Innovation Center for Diagnosis and Treatment of Infectious Diseases, Tsinghua University, Beijing 100084, China; wliu198907@163.com; 8 School of Biomedical Engineering, Faculty of Engineering and IT, University of Technology Sydney, Ultimo, NSW 2007, Australia; Jiaojiao.Li@uts.edu.au; 9 Savaid Medical School, University of Chinese Academy of Sciences, Beijing 100049, China

**Keywords:** embryonic stem cells, mesenchymal stem cells, osteoarthritis, cell therapy, tissue repair

## Abstract

Mesenchymalstem cell (MSC)-based therapy is being increasingly explored in preclinical and clinical studies as a regenerative method for treating osteoarthritis (OA). However, the use of primary MSCs is hampered by a number of limitations, including donor heterogeneity and inconsistent cell quality. Here, we tested the therapeutic potential of embryonic stem cell-derived MSCs (ES-MSCs) in anOA rat model. ES-MSCs were generated and identified by morphology, trilineage differentiation and flow cytometry. Sprague Dawley rats were treated with either a single dose (10^6^ cells/rat) of ES-MSCs or with three doses spaced one week apart for each dose, starting at four weeks after anterior cruciate ligament transectionto induce OA. Cartilage quality was evaluated at 6 and 10 weeks after treatment with behavioral analysis, macroscopic examination, and histology. At sixweeks after treatment, the groups treated with both single and repeated doses of ES-MSCs had significantly better modified Mankin scores and International Cartilage Repair Society (ICRS) macroscopic scores in the femoral condyle compared to the control group. At 10 weeks after treatment, the repeated doses group had a significantly better ICRS macroscopic scores in the femoral condyle compared to the single dose and control groups. Histological analysis also showed more proteoglycan and less cartilage loss, along with lower Mankin scores in the repeated doses group. In conclusion, treatment with multiple injections of ES-MSCs can ameliorate OA in a rat model. TheES-MSCs have potential to be considered as a regenerative therapy for OA, and can provide an infinite cellular source.

## 1. Introduction

Osteoarthritis (OA) is the most prevalent chronic degenerative joint disease worldwide and is associated with a severe health burden for patients and communities [1,2]. OA is defined by structural damage to the joint, including cartilage destruction, subchondral bone sclerosis, and osteophyte formation [3]. Although a range of clinical treatments currently exists for OA, including non-pharmacological, pharmacological, and surgical approaches, all of these can only provide transient relief of symptoms, such as pain, but have relatively small effects in slowing the progression of disease [4,5]. To date, no effective disease-modifying therapy targeting the complicated chronic pathology of OA has been developed [6].

As multipotent progenitor cells, mesenchymal stem cells (MSCs) have the ability to differentiate into multiple lineages of mesenchymal tissue, including bone, cartilage, and fat [7]. MSCs are considered adult stem cells and have been isolated from a variety of tissues, including bone marrow, synovium, dental pulp, skeletal muscle, bone, adipose tissue, placenta, and umbilical cord [8]. It has been reported that MSCs derived from different sources have variable proliferation and differentiation capacity [9], as well as therapeutic potential [10]. Therefore, before implementing new types or sources of MSCs for clinical therapy to treat diseases such as OA, it is essential that their safety and efficacy are investigated through preclinical studies using disease-relevant animal models.

MSC injections have shown promising effects in symptomatic relief and structural repair of the joint in OA animal models and in clinical trials of knee OA [11,12]. The beneficial effects achieved by intra-articular injections of MSCs into the OA joint are thought to be the result of their anti-inflammatory and paracrine functions, which can act to inhibit disease processes [13]. Previously, we have demonstrated that a single injection of MSCs can have temporary effects on slowing the progression of cartilage degeneration in an OA rat model, but their long-term therapeutic benefits were limited [14]. It is often difficult to achieve long-term beneficial effects in an OA joint from a single MSC injection due to extensive cell death or cell loss from the lesion site following injection. One potential method to address this problem is to increase the survival, retention, and function of MSCs by injecting them through a delivery vehicle. For instance, we previously showed that MSCs delivered through 3D microscopic hydrogels could achieve longer term therapeutic effects in a rat OA model, even when a reduced cell dose was used [15]. Nevertheless, such delivery methods require additional prior processing of MSCs, as well as encapsulation and possibly pre-culturing of the MSCs in the delivery vehicle before injection, which may lead to a more extended or complex preparation and limited shelf life. An alternative method to ensure a sufficiently high concentration of MSCs in the OA joint to achieve long-term therapeutic effects is to inject multiple doses of MSCs [16].

Although MSCs derived from adult tissue sources, most commonly bone marrow, adipose tissue, and umbilical cord, can be accessed and isolated relatively easily, their large-scale implementation in clinical therapy may be limited. The recovery rate of purified populations of MSCs from primary adult tissues is still relatively low, which necessitates extensive expansion prior to use in clinical therapies, particularly if multiple doses are required. However, adult MSCs typically have limited self-renewal capacity and a finite lifespan, leading to progressive loss of function with continued expansion. This may lead to adverse effects such as in vitro acquired genomic instability and reactivation of immunogenicity [17].Other common issues of primary MSCs further complicate the matter, such as heterogeneity of cells arising from different donors and tissue sources, and inconsistent cell quality [18]. Recently, embryonic stem cell-derived MSCs (ES-MSCs) have been tested in preclinical studies for their therapeutic effects in a variety of diseases to try and overcome the limitations experienced with primary MSCs [19,20,21]. However, the consistency, safety, and clinical applicability of ES-MSCs is still limited due to the common use of xenogeneic components during culture expansion and maintenance, such as fetal bovine serum (FBS) and murine feeder cells [22].

We previously reported the generation of clinical-grade human ES-MSCs produced under good manufacturing practice (GMP) requirements, which were applied for the in vivo treatment of lung injury and fibrosis [23]. These clinical-grade ES-MSCs have highly consistent quality, and have demonstrated unique abilities in modulating the host immunity and regulating matrix production compared to adult MSCs. In this study, we draw on the high proliferative ability of the clinical-grade ES-MSCs to provide a highly consistent population of MSCs to enable multiple doses. We determined the therapeutic potential of the clinical-grade ES-MSCs when applied to treat knee OA in xenogeneic recipients. In a rat model of knee OA, we compared the efficacy of three injections of ES-MSCs to a single injection and a blank control, through behavioral analysis, macroscopic examination, and histology.

## 2. Results

### 2.1. Generation and Identification of ES-MSCs

**Figure 1:**
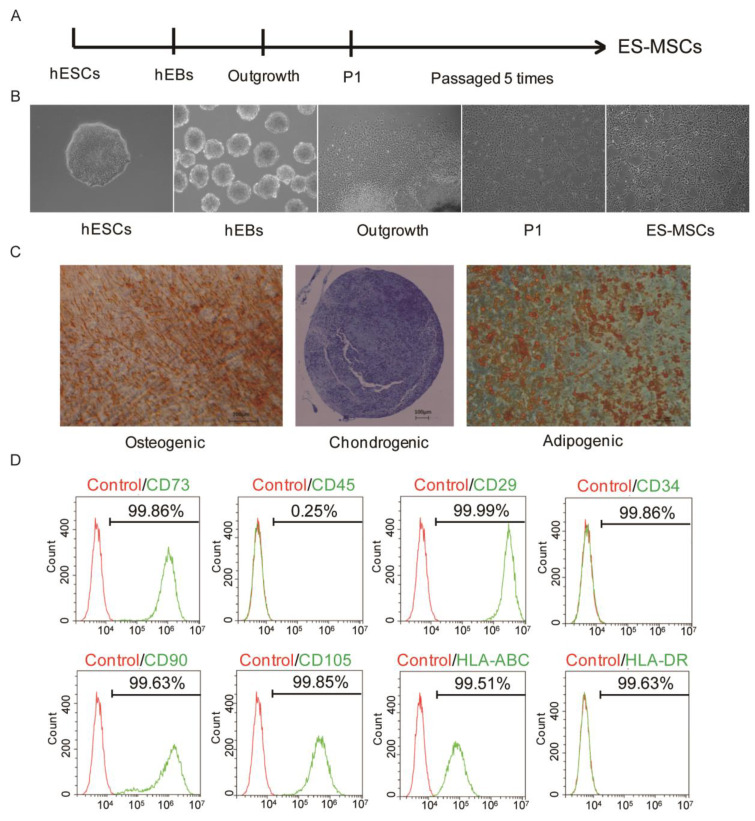
Derivation and identification of embryonic stem cell-derived MSCs (ES-MSCs). (**A**) Derivation protocol ofES-MSCsfrom hESCs. (**B**) Representative morphology of cells at different stages of ES-MSC derivation, observed by phase contrast microscopy. (**C**) Representative staining of ES-MSCs using Alizarin Red S, Oil red O, and Alcian Blue after being induced to undergo osteogenic, adipogenic, and chondrogenic differentiation, respectively. (**D**) Expression of MSC-specific surface markers by ES-MSCs, determined by flow cytometry.

In this study, ES-MSCs were generated by passaging cells that were migrating outwards from hEBs [23] (Figure 1A).Microscopy images showed that the ES-MSCs had spindle-shaped, fibroblast-like morphology, which was distinctly different from the rounded morphology of hESCs (Figure 1B). ES-MSCs displayed the ability to undergo trilineage differentiation into mesenchymal tissues, as shown by Alizarin Red S staining for osteogenesis, Oil red O staining for adipogenesis, and Alcian Blue staining for chondrogenesis (Figure 1C). The surface marker profile of ES-MSCs was also confirmed by flow cytometry analysis, including the expression of CD73, CD29, CD90, CD105, CD45, CD34, HLA-DR, and HLA-ABC (Figure 1D).The ES-MSCs therefore displayed similar morphological, phenotypic and functional characteristics as primary MSCs.

### 2.2. Behavioral Analysis of OA Rats

**Figure 2:**
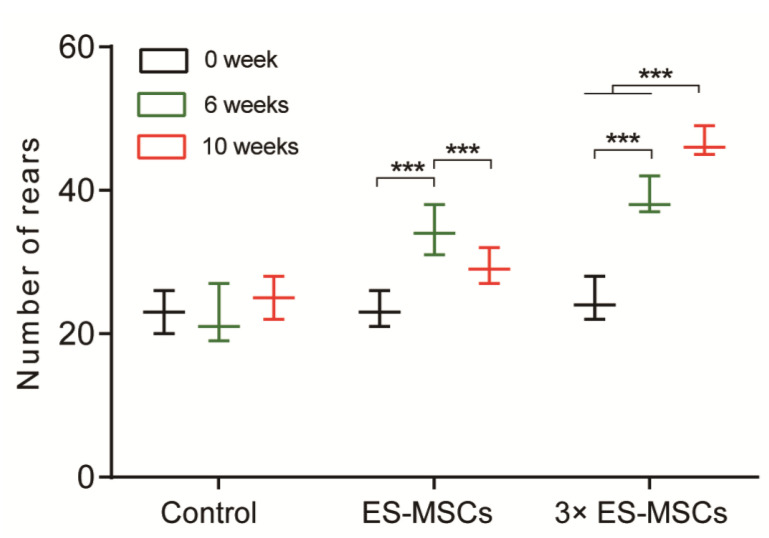
Behavioral analysis. The number of rears performed by animals in the control, ES-MSCs and 3×ES-MSCs groups just before, 6 weeks after, and 10 weeks after the first injection. *** *p* < 0.001.

Rats with induced OA were injected with PBS in the control group, a single dose of cells in the ES-MSCs group, and three doses of cells each spaced one week apart in the 3×ES-MSCs group. The number of rears performed by animals in each group at 6 and 10 weeks following the first injection, shortly before euthanasia were measured (Figure 2). Rearing in the control group showed a non-significant rise from before injection to 10 weeks after injection. The number of rears in the ES-MSCs group peaked at 6 weeks, but dropped significantly at 10 weeks. In contrast, the number of rears in the 3×ES-MSCs group increased significantly at both 6 and 10 weeks compared to the previous time point. In addition, the 3×ES-MSCs group achieved the highest number of rears compared to the other two groups at both 6 and 10 week time points. These results suggested that repeated injections of ES-MSCs were more effective at long-term relief of joint pain in the OA rat model compared to a single injection.

### 2.3. Macroscopic Evaluation of the Knee Joint in OA Rats

**Figure 3:**
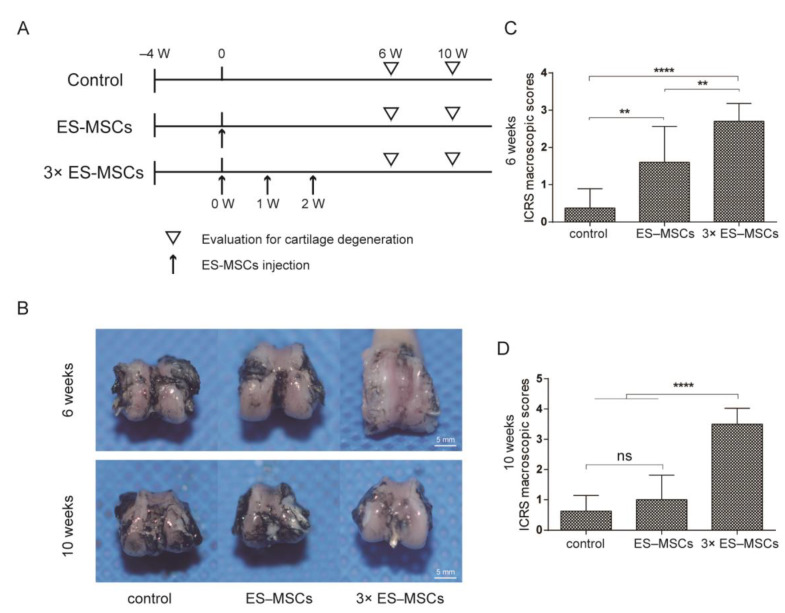
Macroscopic evaluation of the knee joint. (**A**) Schematic of thein vivo study timeline. (**B**) Representative macroscopic features of the femoral condyle as shown by India ink from three specimens per group at 6 and 10 weeks after the first injection. (**C**) ICRS macroscopic scores of the femoral condyle for all groups at 6 after the first injection. (**D**) ICRS macroscopic scores of the femoral condyle for all groups at 10 weeks after the first injection. Error bars represent 95% confidence intervals (CI). ** *p* < 0.01, **** *p* < 0.0001, ns = no significance.

Following intra-articular injection of rat OA knee joints, macroscopic observations of the distal femur were compared among the three groups injected with PBS, or ES-MSCs as a single dose or three doses, at 6 and 10 weeks following the first injection (Figure 3A). At 6 weeks, the joint surface in the control group showed marked macroscopic signs of OA progression, including cartilage surface roughness and osteophyte formation, compared to the relatively well-preserved cartilage surface in the ES-MSCs and 3×ES-MSCs groups (Figure 3B). However, at 10 weeks, the joint surface in both the control and ES-MSCs groups showed significant OA progression and cartilage deterioration, with only the 3×ES-MSCs group displaying a preserved cartilage surface. The International Cartilage Repair Society (ICRS) macroscopic scores reflected similar trends (Figure 3C,D). ICRS scores in the ES-MSCs group were significantly higher than the control group at 6 weeks, but this was no longer the case at 10 weeks. For the 3×ES-MSCs group, its ICRS scores were significantly higher than the other two groups at both 6 and 10 weeks (6 weeks: F = 181.7, *p* < 0.001; control vs. ES-MSCs: mean difference (MD) = 1.08, 95%confidence index (CI) (0.30–1.85), *p* < 0.01; control vs. 3×ES-MSCs: MD = 5.48, 95%CI (4.70–6.25), *p* < 0.001; ES-MSCs vs. 3×ES-MSCs: MD = 4.40, 95%CI (3.67–5.14), *p* < 0.001. At 10 weeks: F = 150.8, *p* <0.001; control vs. 3×ES-MSCs: MD = 7.53, 95%CI (6.32–8.73), *p* < 0.001; ES-MSCs vs. 3×ES-MSCs: MD = 6.50, 95%CI (5.36–7.64), *p* < 0.001). These findings aligned with the observations from behavioral analysis, and suggested that multiple injections of ES-MSCs were effective in suppressing macroscopic changes in OA joints during the early and late stages after cell therapy treatment.

### 2.4. Histological Analysis of the Knee Joint in OA Rats

**Figure 4:**
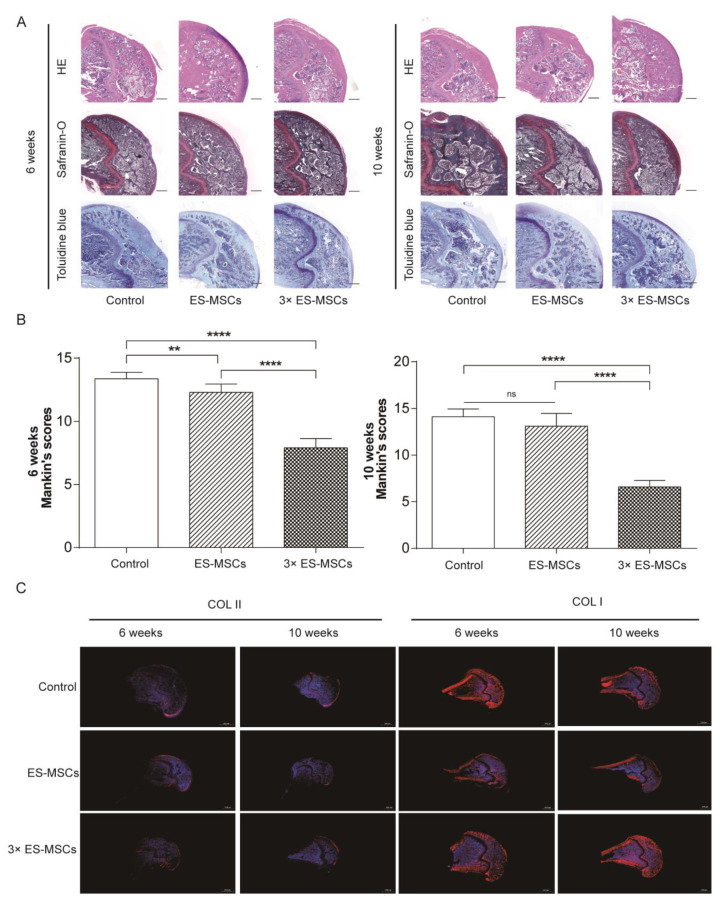
Histological analysis of the knee joint. (**A**) Histological images showing HE, safranin O and toluidine blue staining of the medial femoral condyle in all groups, at 6 and 10 weeks after the first injection. (**B**) The corresponding modified Mankin scoresfor all groups. (**C**) Immunofluorescence staining for collagen types I and II in all groups at 6 and 10 weeks after the first injection.Error bars represent 95% confidence intervals (CI).** *p* < 0.01, **** *p* < 0.001, ns = no significance.

Representative histological images of the medial femoral condyle are shown for all groups at 6 and 10 weeks post-treatment, stained by hematoxylin and eosin (HE), safraninO, and toluidine blue (Figure 4A). At 6 weeks, the articular cartilage in the control group showed surface irregularity, loss of cellularity and reduced area of safranin O staining, indicating significant OA changes. In contrast, articular cartilage in the ES-MSCs and 3×ES-MSCs groups showed abundant proteoglycan and reduced cartilage loss, without significant signs of OA progression. At 10 weeks, the histological features of preserved joint structure were similar compared to at 6 weeks in the 3×ES-MSCs group. However, the ES-MSCs group showed reduced joint integrity compared to at 6 weeks and displayed features of OA progression similar to those in the control group, including cartilage surface irregularities, loss of cellularity and tidemark integrity, and significant proteoglycan loss.

Modified Mankin scores were used to evaluate the severity of cartilage degeneration from histological sections in all groups (Figure 4B). At 6 weeks, modified Mankin scores for the ES-MSCs and 3×ES-MSCs groups were significantly reduced compared to the control group (F = 24.30, *p* < 0.001; control vs. ES-MSCs: MD = −1.23, 95%CI (−2.06 to −0.40), *p* < 0.01; control vs. 3×ES-MSCs: MD = −2.33, 95%CI (−3.16 to −1.49), *p* < 0.001; control vs. ES-MSCs: MD = −1.23, 95%CI (−2.06 to −0.40), *p* < 0.01; ES-MSCs vs. 3×ES-MSCs: MD = −1.10, 95%CI (−1.89 to −0.32), *p* < 0.01). At 10 weeks, modified Mankin scores were no longer significantly different between the ES-MSCs and control groups, while the 3×ES-MSCs group still had significantly lower scores than the other two groups (F = 0.66, *p* = 0.53; control vs. ES-MSCs: MD = −0.38, 95%CI (−1.14 to 0.39), *p* > 0.05; control vs. 3×ES-MSCs: MD = −2.88, 95%CI (−3.64 to −2.11), *p* < 0.001; ES-MSCs vs. 3×ES-MSCs: MD = −2.50, 95%CI(−3.21 to −1.78), *p* < 0.001). The histological results were matched by immunofluorescence staining for collagen type II and collagen type I in the joint sections (Figure 4C). These findings collectively indicate that while a single dose of ES-MSCs could be effective at preserving joint structure and retarding OA progression over the shorter-term, repeated injections of ES-MSCs were necessary to produce sustained beneficial effects in the long-term.

## 3. Discussion

OA is often associated with significant pain that greatly limits the mobility of patients, leading to substantial reductions in the quality of life and commitment of healthcare resources for ongoing disease management [24]. The lack of effective therapies to stop OA progression has propelled the development of new interventions, most recently approaches based on regenerative medicine such as cell and gene therapies. However, our recent reviews on the current evidence of using intra-articular injections of MSCs to treat knee OA in animal studies and in clinical trials showed inconclusive benefits, and indicated low confidence of recommending this as a reliable and effective long-term therapy for OA [11,12]. Our previous study in a rat model suggested that a single injection of primary MSCs could only have temporary, early-stage effects on retarding the progression of OA in the knee joint [14]. This pointed to the possibility that a critical concentration of cells needed to be maintained in the OA knee joint, particularly during the early stages of treatment, to provide sustained therapeutic effects in preserving the joint structure. Increased cell concentrations can be easily achieved by administering multiple injections of MSCs, but this approach can be hampered in clinical translation by the large number of total cells required.

Although primary MSCs derived from adult tissues have already been tested in various clinical studies in the treatment of knee OA, their widespread clinical implementation is limited by several challenges, including limited cell numbers and self-renewal capacity, donor heterogeneity, and inconsistent cell quality [25]. Although secretomes were considered a promising option to exploit primary MSCs properties to address OA, their standardizations and the effects of their components in the complex in microenvironment of OA were still lacking understanding [26]. These issues are exacerbated if either high doses of cells, repeated doses, or both, are required to produce sustained therapeutic effects. To address this problem, we developed a new ES-MSC cell population with unique ability to maintain long-term proliferation without losing functional and phenotypic characteristics, as well as capacity for immunomodulation and regulation of extracellular matrix production which supplement their regenerative functions [23]. Although ES-derived MSCs could be generated through various other protocols, no such cell products have yet been translated into clinical application. This is likely due to the common concerns and stigmas surrounding human ESCs, such as immunogenicity from allogeneic transplantation, and possible tumorigenicity of incompletely differentiated cells. With these concerns in mind, we developed the clinical-grade ES-MSCs used in this study, and have subjected them to testing through a series of biosafety-related experiments. We also showed that there was no teratoma formation observed after ES-MSCs injection into mice. Further, we confirmed that the ES-MSCs were immunoprivileged without allogeneic immune responses. Such studies are essential for demonstrating that these ES-MSCs are suitable for use in human therapy according to the “Guidelines for Human Somatic Cell Therapies and Quality Control of Cell-based Products” [27].After the previous safety evaluations, testing in physiologically relevant animal models are necessary to evaluate the therapeutic effects of ES-MSCs in the treatment of different diseases. In this study, we applied multiple doses of ES-MSCs to test their effects in ameliorating disease progression following OA induction in a rat model, compared to a single dose and to untreated animals. We selected 6 and 10 weeks after the first injection as time points for analysis as these allow the observation of early- and late-stage changes, respectively, in the progression of OA in rat models [11].

The injections were administered at 4 weeks following OA induction surgery, which ensured that OA disease progression was already well established by the time that the treatments were applied. At the early stages (6 weeks) following treatment, the behavioral, macroscopic, and histological analyses collectively indicated that the groups injected with ES-MSCs, whether as a single dose or in multiple doses, were both highly effective at symptom relief and protection of joint structure in OA rats compared to untreated controls. Notably, compared to the structural disorganization in the joint and significant OA progression seen in the control group, the two ES-MSCs groups both showed relatively normal joint features at 6 weeks, with very mild irregularity in the surface layer, a normal distribution of cells, appropriate cartilage thickness, and consistent staining for articular cartilage. These results suggested that a single injection of ES-MSCs was able to exert significant short-term effects in protecting the joint from degradation during OA progression, which matched the findings of other studies testing different sources of MSCs in rat OA models over similar time periods [14]. Nevertheless, even at the earlier time point of 6 weeks, multiple injections of ES-MSCs were shown to provide greater improvements in macroscopic and histological findings compared to a single injection.

At later stages (10 weeks) following treatment, the benefits provided by a single injection of ES-MSCs were significantly reduced, with both macroscopic and histological analysis scores declining to similar levels as the control. In contrast, multiple injections of ES-MSCs continued to provide significant therapeutic effects in hampering OA progression, and these therapeutic effects were greater at 10 weeks compared to at 6 weeks for all of the analyses conducted. These results matched the findings of another study that applied periodic injections of synovial MSCs in rats, which inhibited OA progression to a greater extent than a single injection, which was thought to be due to a sustained secretion of paracrine factors [28]. Using multiple injections of MSCs to treat OA may provide benefits from different aspects. First, the number of MSCs in the joint could decrease rapidly, possibly within a matter of days following a single injection [29,30], which would leave insufficient numbers to counteract the long-term pathological progression of OA. Second, the surviving MSCs could adversely respond to the diseased joint environment [31], particularly with increased time of exposure. This is because MSCs have an intrinsic ability to sense and respond to changes in the external environment, including a wide range of biophysical and biochemical factors, and consequently change their behavior. When placed within a diseased OA environment, a single dose of MSCs might rapidly cease their secretion of beneficial factors. By providing multiple doses of MSCs at appropriate intervals, the supplementation of immunomodulatory and trophic factors by MSCs into the OA joint could be sustained, which might regulate or possibly reverse the pathological joint environment to provide long-term therapeutic benefits. This study demonstrates the use of a stable, reliable, and consistent source of ES-MSCs to provide multiple doses of cells for treating OA, which provides promise for satisfying the needs for large amounts of cells when considering clinical implementation. Future studies investigating the long-term therapeutic effects of multiple injections of ES-MSCs, using larger animal models, different concentrations of cells and running over physiologically-relevant time periods will be necessary to further characterize the clinical relevance of this approach.

## 4. Materials and Methods

### 4.1. Experiment Design

**Figure 5:**
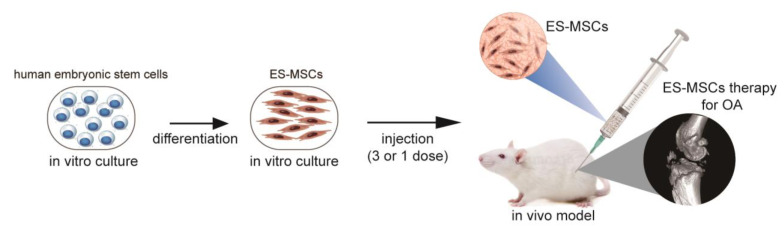
Schematic illustration of the study design. ES-MSCs were generated and identified. Knee joints in a rat model of OA were treated with ES-MSCs (single dose or three doses) in 100 μL PBS 4 weeks after surgery.

ES-MSCs were generatedin vitro and assessed for their phenotypic and functional characteristics, as previously described [23]. The ES-MSCs were injected into the knee joint of rats, as three separate doses or a single dose, at 4 weeks after surgery-induced OA. Macroscopic, behavioral, and histological analyses were conducted at 6 and 10 weeks after the first injection (Figure 5).

### 4.2. Derivation of ES-MSCs

The ES-MSCs were generated by passaging cells that were migrating outwards from human embryoid bodies (hEBs), using serum-free reagents. Clinical-grade ES-MSCs were prepared and expanded as described previously [23], using the clinical human embryonic stem cell (hESC) line QCTS-hESC-2. The hESCs were maintained in Essential 8 basal medium (E8), before dissociation into small clumps to form hEBs for 5 days. ThehEBs were then cultured for another 14 days. Outgrowth cells from thehEBs, which were considered P1 cells, were passaged continuously in ES-MSC medium [23]. After 5 passages, ES-MSCs were derived, which were harvested for characterization or expanded for use in thein vivo study.

### 4.3. Flow Cytometry

The ES-MSCs were digested from flasks and suspended in phosphate-buffered saline (PBS). The anti-human antibodies CD34, CD45, HLA–DR, HLA–ABC, CD90, CD29, CD73, and CD105 (BioLegend, CA, USA) were used for flow cytometry according to the manufacturer’s instructions. Antibody incubations were conducted on ice for 30 min. The ES-MSCs were then washed and resuspended in PBS for flow cytometry (BD LSRFortessa SORP, NJ, USA).

### 4.4. Trilineage Differentiation Assay

Osteogenic, adipogenic, and chondrogenic differentiation experiments were performed following the instructions of the human mesenchymal stem cell functional identification kit (R&D systems, SC006, MN, USA). For osteogenic differentiation, 4.2 × 10^3^ cells were seeded per well in 96-well plates. When cells reached 50–70% confluency, the medium was replaced with osteogenic differentiation medium and kept for 3 weeks. To assess osteogenic differentiation, Alizarin Red S (SigmaAldrich, A5533, MO, USA) staining was performed for the calcium-rich extracellular matrix. For adipogenic differentiation, cells were seeded into a 24-well plate at the density of 3.7 × 10^4^ cells/well, and maintained in culture medium until 100% confluency. Then, cells were cultured in adipogenic differentiation medium for 3 weeks. Lipid droplets of the resultant differentiated cells were detected using Oil red O (SigmaAldrich, O0625, MO, USA) staining. For chondrogenic differentiation, 2.5 × 10^5^ cells resuspended in chondrogenic differentiation medium were centrifuged for 5 min at 200× *g* in a 15 mL conical tube (Corning, NY, USA). Then, cells were cultured for 3 weeks. After 3 weeks, chondrogenic pellet was harvested and fixed in 4% paraformaldehyde (Aladdin Chemical Co., Ltd., C104190, Shanghai, China). Cryosectioning was performed by OTF Cryostat (Leica, Wetzlar, Germany) and 10 µm sections were stained with Alcian Blue (Sigma-Aldrich, A5268, MO, USA) staining.

### 4.5. OA Animal Model

Twenty-eight Sprague Dawley rats (10 weeks old; mean weight 160 g) were randomly divided into three groups: control group (*n* = 8), ES-MSCs group (*n* = 10), and 3×ES-MSCs group (*n* = 10). Bilateral knee OA was induced by anterior cruciate ligament (ACL) transection. Briefly, the rats were anesthetized, and surgery was performed to transect the ACL. After surgery, each rat was administered penicillin once per day for the first three days. The control group was injected with 100μLPBS into the intra-articular spaceof the bilateral knee joint at 4 weeks after surgery. For the ES-MSCs group, rat knees were injected with a single dose of ES-MSCs (10^6^ cells/joint) in 100μLPBSat 4 weeks after surgery. For the 3×ES-MSCs group, ratkneeswere injected with three doses of ES-MSCs (10^6^ cells/joint) in 100 μL PBS, one week apart for each dose.

#### 4.5.1. Behavioral Analysis

Rearing (standing on rear limbs) in animals was counted before the first injection, and at 6 and 10 weeks after the first injection [32,33]. A box (70 cm × 30 cm × 30 cm) was placed in a room without noise. The bottom of the box was covered with foam stamp pads. The four sides of the box were covered with white paper. The paws of the rats were stained with ink from thefoam stamp pads. The rats left pawprints on the white paper when they stood on their hindlimbs and touched the walls of the box with their forelimbs. To distinguish rearing from incomplete standing actions, the reliable rears were defined as being at a height of at least 5 cm from the bottom. Each rat was left in the box for 10 min. The number of rears was counted manually.

#### 4.5.2. Macroscopic Examination

The rats were sacrificed at 6 and 10 weeks after the first injection.The surface of the distal femur was exposed, and 2 mL ofIndia ink was injected onto the distal femur with a syringe. After 2 min, the surface was washed with saline, and the staining pattern of the distal femur was examined macroscopically. Further, macroscopic evaluation was conducted according to the cartilage repair assessment instrument of the International Cartilage Repair Society (ICRS) [34]. The assessment was performed based on macroscopic examination of the cartilage surface, which was scored from 4 to 0 (4: Intact smooth surface. 3: Fibrillated surface. 2: Small, scattered fissures, or cracks. 1: Several, small, or few but large fissures. 0: Total degeneration of surface area).

#### 4.5.3. Histology and Immunohistochemistry

The knee joints were harvested and fixed in 4% paraformaldehyde overnight. Decalcification was conducted in 4% ethylene diamine tetraacetic acid (EDTA) for one month, with the decalcifying solution changed every 3 days. Decalcified joints were embedded in paraffin and used to cut 4 μm sections of the medial femoral condyle. The sections were stained using hematoxylin and eosin (HE), safranin O, and toluidine blue.

The severity of cartilage degeneration was assessed in histological sections using the modified Mankin score [35], which was scored based on: (1) surface integrity (score 0–10), (2) cellularity (score 0–4), (3) cell clones (score 0–4), and (4) Safranin O staining (score 0–5). A higher score indicated a greater level of cartilage degeneration.

Immunohistochemical staining was conducted using the histological sections. Briefly, after deparaffinization, sections were incubated with 0.3% hydrogen peroxide for 30 min and treated with hyaluronidase for 60 min. The sections were then incubated with COL-I or COL-II monoclonal antibodies (Santa Cruz Biotechnology, Dallas, TX, USA). All antibody dilutions were made in PBS. After an overnight reaction with the primary antibody at 4 °C, sections were incubated with labelled polymer-HRP anti-mouse IgG at room temperature for 30 min.

### 4.6. Statistical Analysis

All data were expressed as mean ± standard deviation. After testing for homogeneity of variances, one-way analysis of variance (ANOVA) followed by Tukey’s post hoc multiple comparisons test were used to determine significant differences between groups. SPSS 11.0 (IBM Company, Chicago, IL, USA) was used to perform statistical analyses, and values of *p* < 0.05 were considered statistically significant.

## 5. Conclusions

The administration of ES-MSCs can have significant therapeutic effects in decelerating the progression of OA in a rat model, as suggested by behavioral, macroscopic, and histological analysis. While a single injection of cells provided only short-term improvements, multiple injections demonstrated superior therapeutic effects that were sustained over both the short- and long-term. The ES-MSCs could provide a theoretically infinite cellular source for therapeutic interventions in OA, that would not be hampered by the limited proliferative ability of primary MSCs to supply sufficient cell numbers for multiple injections.

## Data Availability

The data presented in this study are available on request from the corresponding author. The data are not publicly available due to data protection protocol among researchers.

## Sample Availability

Not available.

## References

[1] Vos T., Flaxman A.D., Naghavi M., Lozano R., Michaud C., Ezzati M., Shibuya K., Salomon J.A., Abdalla S., Aboyans V. (2012). Years lived with disability (YLDs) for 1160 sequelae of 289 diseases and injuries 1990–2010: A systematic analysis for the Global Burden of Disease Study 2010. Lancet.

[2] Hunter D.J., Schofield D.J., Callander E.J. (2014). The individual and socioeconomic impact of osteoarthritis. Nat. Rev. Rheumatol..

[3] Riordan E., Little C., Hunter D. (2014). Pathogenesis of post-traumatic OA with a view to intervention. Best Pract. Res. Clin. Rheumatol..

[4] de l’Escalopier N., Anract P., Biau D. (2016). Surgical treatments for osteoarthritis. Ann. Phys. Rehabil. Med..

[5] Filardo G., Kon E., Longo U.G., Madry H., Marchettini P., Marmotti A., Van Assche D., Zanon G., Peretti G.M. (2016). Non-surgical treatments for the management of early osteoarthritis. Knee Surg. Sports Traumatol. Arthrosc..

[6] Sellam J., Berenbaum F. (2010). The role of synovitis in pathophysiology and clinical symptoms of osteoarthritis. Nat. Rev. Rheumatol..

[7] Pittenger M.F., Mackay A.M., Beck S.C., Jaiswal R.K., Douglas R., Mosca J.D., Moorman M.A., Simonetti D.W., Craig S., Marshak D.R. (1999). Multilineage Potential of Adult Human Mesenchymal Stem Cells. Science.

[8] Kim J.-Y., Jeon H.B., Yang Y.S., Oh W., Chang J.W. (2010). Application of human umbilical cord blood-derived mesenchymal stem cells in disease models. World J. Stem Cells.

[9] Hass R., Kasper C., Böhm S., Jacobs R. (2011). Different populations and sources of human mesenchymal stem cells (MSC): A comparison of adult and neonatal tissue-derived MSC. Cell Commun. Signal..

[10] Liao H.T., Chen C.T. (2014). Osteogenic potential: Comparison between bone marrow and adipose-derived mesenchymal stem cells. World J Stem Cells.

[11] Xing D., Kwong J., Yang Z., Hou Y., Zhang W., Ma B., Lin J. (2018). Intra-articular injection of mesenchymal stem cells in treating knee osteoarthritis: A systematic review of animal studies. Osteoarthr. Cartil..

[12] Xing D., Wang Q., Yang Z., Hou Y., Zhang W., Chen Y., Lin J. (2017). Mesenchymal stem cells injections for knee osteoarthritis: A systematic overview. Rheumatol. Int..

[13] van Buul G.M., Villafuertes E., Bos P.K., Waarsing J.H., Kops N., Narcisi R., Weinans H., Verhaar J.A., Bernsen M.R., van Osch G.J. (2012). Mesenchymal stem cells secrete factors that inhibit inflammatory processes in short-term osteoarthritic synovium and cartilage explant culture. Osteoarthr. Cartil..

[14] Xing D., Wu J., Wang B., Liu W., Liu W., Zhao Y., Wang L., Li J.J., Liu A., Zhou Q. (2020). Intra-articular delivery of umbilical cord-derived mesenchymal stem cells temporarily retard the progression of osteoarthritis in a rat model. Int. J. Rheum. Dis..

[15] Xing D., Liu W., Wang B., Li J.J., Zhao Y., Li H., Liu A., Du Y., Lin J. (2020). Intra-articular Injection of Cell-laden 3D Microcryogels Empower Low-dose Cell Therapy for Osteoarthritis in a Rat Model. Cell Transplant..

[16] Gonzalo-Gil E., Pérez-Lorenzo M.J., Galindo M., De La Guardia R.D., Lopez-Millan B., Bueno C., Menéndez P., Pablos J.L., Criado G. (2016). Human embryonic stem cell-derived mesenchymal stromal cells ameliorate collagen-induced arthritis by inducing host-derived indoleamine 2,3 dioxygenase. Arthritis Res..

[17] Rodriguez R., Rosu-Myles M., Aráuzo-Bravo M., Horrillo A., Pan Q., Gonzalez-Rey E., Delgado M., Menéndez P. (2014). Human Bone Marrow Stromal Cells Lose Immunosuppressive and Anti-inflammatory Properties upon Oncogenic Transformation. Stem Cell Rep..

[18] Andrzejewska A., Lukomska B., Janowski M. (2019). Concise review: Mesenchymal stem cells: From roots to boost. Stem Cells.

[19] Hawkins K.E., Corcelli M., Dowding K., Ranzoni A.M., Vlahova F., Hau K.-L., Hunjan A., Peebles D., Gressens P., Hagberg H. (2018). Embryonic Stem Cell-Derived Mesenchymal Stem Cells (MSCs) Have a Superior Neuroprotective Capacity Over Fetal MSCs in the Hypoxic-Ischemic Mouse Brain. Stem Cells Transl. Med..

[20] Kim C.-H., Lim C.-Y., Lee J.-H., Kim K.C., Ahn J.Y., Lee E.J. (2019). Human Embryonic Stem Cells-Derived Mesenchymal Stem Cells Reduce the Symptom of Psoriasis in Imiquimod-Induced Skin Model. Tissue Eng. Regen. Med..

[21] Abbaszadeh H., Ghorbani F., Derakhshani M., Movassaghpour A., Yousefi M. (2020). Human umbilical cord mesenchymal stem cell-derived extracellular vesicles: A novel therapeutic paradigm. J. Cell. Physiol..

[22] Hematti P. (2010). Human Embryonic Stem Cell-Derived Mesenchymal Progenitors: An Overview. Adv. Struct. Saf. Stud..

[23] Wu J., Song D., Li Z., Guo B., Xiao Y., Liu W., Liang L., Feng C., Gao T., Chen Y. (2020). Immunity-and-matrix-regulatory cells derived from human embryonic stem cells safely and effectively treat mouse lung injury and fibrosis. Cell Res..

[24] Hawker G.A., Croxford R., Bierman A.S., Harvey P.J., Ravi B., Stanaitis I., Lipscombe L.L. (2014). All-Cause Mortality and Serious Cardiovascular Events in People with Hip and Knee Osteoarthritis: A Population Based Cohort Study. PLoS ONE.

[25] Trivedi A., Miyazawa B., Gibb S., Valanoski K., Vivona L., Lin M., Potter D., Stone M., Norris P.J., Murphy J. (2019). Bone marrow donor selection and characterization of MSCs is critical for pre-clinical and clinical cell dose production. J. Transl. Med..

[26] D’Arrigo D., Roffi A., Cucchiarini M., Moretti M., Candrian C., Filardo G. (2020). Faculty Opinions recommendation of Secretome and extracellular vesicles as new biological therapies for knee osteoarthritis: A systematic review. J. Clin. Med..

[27] Petricciani J., Hayakawa T., Stacey G.N., Trouvin J.-H., Knezevic I. (2017). Scientific considerations for the regulatory evaluation of cell therapy products. Biologicals.

[28] Ozeki N., Muneta T., Koga H., Nakagawa Y., Mizuno M., Tsuji K., Mabuchi Y., Akazawa C., Kobayashi E., Matsumoto K. (2016). Not single but periodic injections of synovial mesenchymal stem cells maintain viable cells in knees and inhibit osteoarthritis progression in rats. Osteoarthr. Cartil..

[29] ter Huurne M., Schelbergen R., Blattes R., Blom A., de Munter W., Grevers L.C., Jeanson J., Noël D., Casteilla L., Jorgensen C. (2012). Antiinflammatory and chondroprotective effects of intraarticular injection of adipose-derived stem cells in experimental osteoarthritis. Arthritis Rheum..

[30] Horie M., Choi H., Lee R.H., Reger R.L., Ylostalo J., Muneta T., Sekiya I.C., Prockop D.J. (2012). Intra-articular injection of human mesenchymal stem cells (MSCs) promote rat meniscal regeneration by being activated to express Indian hedgehog that enhances expression of type II collagen. Osteoarthr. Cartil..

[31] Murphy M.B., Moncivais K., Caplan A.I. (2013). Mesenchymal stem cells: Environmentally responsive therapeutics for regenerative medicine. Exp. Mol. Med..

[32] Kim S.J., Kim J.E., Lee S.M., Kim S.H., Tatman P., Gee A.O., Kim D.-H., Lee K.E., Jung Y. (2014). Effect of self-assembled peptide–mesenchymal stem cell complex on the progression of osteoarthritis in a rat model. Int. J. Nanomed..

[33] Nagase H., Kumakura S., Shimada K. (2012). Establishment of a novel objective and quantitative method to assess pain-related behavior in monosodium iodoacetate-induced osteoarthritis in rat knee. J. Pharmacol. Toxicol. Methods.

[34] van den Borne M.P., Raijmakers N.J., Vanlauwe J., Victor J., De Jong S.N., Bellemans J., Saris D.B.F. (2007). International Cartilage Repair Society (ICRS) and Oswestry macroscopic cartilage evaluation scores validated for use in Autologous Chondrocyte Implantation (ACI) and microfracture. Osteoarthr. Cartil..

[35] Moody H.R., Heard B.J., Frank C.B., Shrive N.G., Oloyede A. (2012). Investigating the potential value of individual parameters of histological grading systems in a sheep model of cartilage damage: The Modified Mankin method. J. Anat..

